# Comparison of dynamic cyclic fatigue resistance of Reciproc® Blue and WaveOne® Gold after sterilization and/or immersion in sodium hypochlorite

**DOI:** 10.4317/jced.60870

**Published:** 2024-01-01

**Authors:** Néstor Ríos-Osorio, Javier Caviedes-Bucheli, Juan Murcia-Celedón, Cristina Gutiérrez, Diana Sierra-Collazo, Brandon Alvarado-Caicedo, Mariangel Serchiaro-Monsalve, Paola Echavarria-Sarabia, Andrea Leon-Lazzo, Helen Rivera-Rojas, Kathy Castrillón- Ramos, Alfredo Supelano-Gallego, Luisa Bermúdez-Zuluaga, Oscar Jimenez-Peña

**Affiliations:** 1 DDS, MSc. Research Department COC- CICO, Institución Universitaria Colegios de Colombia UNICOC, Bogotá, Colombia; 2 DDS, MSc. Centro de Investigaciones Odontológicas Pontificia Universidad Javeriana Bogotá, Colombia; 3 DDS. Research Department COC- CICO, Institución Universitaria Colegios de Colombia UNICOC, Bogotá, Colombia; 4 DDS. Postgraduate Endodontics Department, Institución Universitaria Colegios de Colombia UNICOC, Bogotá, Colombia; 5 DDS, MSc, PHD. Research Department COC- CICO, Institución Universitaria Colegios de Colombia UNICOC, Bogotá, Colombia

## Abstract

**Background:**

This study aims to compare the cyclic fatigue resistance (CFR) of the Reciproc Blue and WaveOne Gold instruments under a dynamic cyclical fatigue test.

**Material and Methods:**

210 Reciproc Blue “R25” and WaveOne Gold “primary” files were assigned into 7 groups (n =15) for each brand. Groups G: Files were not exposed to NaOCl or sterilization. Groups A and D: files were immersed for 3 minutes in NaOCl 1 and 3 times, respectively. Groups B and E: Files were autoclaved 1 and 3 times, respectively. Groups C and F: files were exposed to both, NaOCl immersion and autoclaving 1 and 3 times, respectively. Subsequently, files underwent a dynamic CFR test. The chemical composition of the files’ surface from Group G was analysed by energy-dispersive X-ray spectroscopy (EDS). Cyclic fatigue resistance time was statistically analysed using 1-way and 2-way analyses of variance (ANOVA) and T-test. A *p*-value ≤0,05 was set to be statistically significant.

**Results:**

There was a significantly higher CFR of RB files than WOG in groups A, B, C, D and G (*p*<0.05). WOG files were superior to RB in group E (*p*>0.05). There were no statistically significant differences between files in group F (*p*>0.05). RB files from groups B, C, D, E and F had significantly lower resistance than new ones (Group G) (*p*<0.05). WaveOne Gold files exposed to 5 % NaOCl immersion in combination with sterilization cycles (Groups C and F) had significantly lower CFR than new ones (*p*<0.05). Reciproc Blue and WaveOne Gold NiTi alloys differed in atomic wt % of carbon, oxygen, nickel and titanium.

**Conclusions:**

The Reciproc Blue files outperformed the WaveOne Gold files in terms of CFR. The Reciproc Blue files were more vulnerable to the cycles of NaOCl immersion or autoclave sterilisation. The combined autoclaving and NaOCl cycles had the most significant impact on the mechanical properties of both files.

** Key words:**Cyclic fatigue, Reciproc blue, Wave one gold, Dynamic test, Simulated channels, Sodium hypochlorite, Sterilization.

## Introduction

One of the primary issues in endodontic practise is the fracture of nickel-titanium (NiTi) rotary instruments. Such a complication can occur mainly by two mechanical deficiencies: (i) torsional failure or (ii) flexural cyclic fatigue (1). Torsional fatigue occurs when the apical part of the instrument gets blocked in the root canal walls while the shank of the file keeps rotating. When the elastic limit of the alloy is exceeded, the fracture is an ineviTable event (2). Flexural cyclic fatigue is caused by repeated tension/compression cycles at the file’s maximum bending point while rotating within a curved root canal until fracture occurs (3).

Several strategies have been proposed to improve the mechanical properties of NiTi instruments, such as electro-polishing treatments, ion implantation, surface modifications and thermal treatments, attempting to improve flexibility and increase cyclic fatigue resistance (CFR) (2,3). In addition, the reciprocating alternating motion kinematics has positively impacted the overall useful life of NiTi endodontic instruments compared to continuous rotation systems (4). The reciprocating dynamic entails a counter-clockwise (CCW) movement aimed to perform the cutting action, followed by a clockwise (CW) movement to release the file from the dentin walls; resulting in lower voltage values on the instruments and reducing cyclic fatigue over time (4).

Reciproc® Blue (VDW, Munich, Germany) and WaveOne® Gold (Dentsply Maillefer, Ballaigues, Switzerland) are two of the main examples of reciprocating systems. Both instruments are manufactured with Control Memory (CM) wire technology. CM-wires are thermo-mechanically treated NiTi alloys, having a lower nickel (Ni) content than conventional NiTi files. CM-wires contain a high martensite content, and in contrast to conventional NiTi, CM-wires do not display super-elasticity (5). The thermal treatment (responsible for exerting a controlled-memory effect on the files) is mainly applied in the martensitic phase (6,7).

Reciproc® Blue (RB) is the second generation of the Reciproc file system. RB instruments have an S-shaped cross-section, 2 cutting edges, and a noncutting tip. Unlike the former Reciproc system, RB files are manufactured by altering the molecular structure through a thermal treatment, which gives the files their blue colour, improved flexibility and high CFR (6).

WaveOne® Gold (WOG) is the latest version of the WaveOne files. The cross-section of WaveOne Gold files is a parallelogram with an 85-degree active cutting edge, with alternate one- and two-point contact. The WaveOne Gold files are fabricated by heating and then slowly cooling the file after production (gold heat treatment), which increases its flexibility and CFR (6).

Endodontic files’ CFR can be directly influenced by a variety of clinical procedure-related variables, such as sterilising cycles and the corrosion brought on by sodium hypochlorite (NaOCl) solutions (8). The corrosion patterns caused by NaOCl can have a negative impact on the mechanical and physical characteristics of NiTi instruments, as it selectively removes nickel from the surface of the instrument and causes micro-pitting, resulting in stress concentrations and crack formation (9). Contrarily, it has been suggested that the temperature generated during sterilization processes could affect crystalline phases in the NiTi alloy, resulting in higher cutting efficacy and improved resistance to fatigue failure (8).

CFR of NiTi rotary files is usually tested with the rotational bending test. According to no. 28 ANSI/ADA specification (American National Standards Institute/American Dental Association), these tests must be performed in a static condition in which the rotary files are allowed to rotate freely in an artificial glass or metal device with different geometric curvature until a fracture occurs (without involving the axial oscillation, commonly used during clinical endodontic instrumentation techniques with NiTi rotary files) (1,10). However, several studies have concluded that dynamic cyclic fatigue tests in which the files are operated with axial oscillation movement simultaneous to the free rotation inside the artificial device provide more reliable results regarding resistance to cyclic fatigue and also better represent the clinical reality (10,11).

In view of the foregoing, the main objective of this study was to compare the CFR of the RB “R25” and WOG “Primary” instruments under a dynamic cyclical fatigue test, after immersion in 5% sodium hypochlorite and/or sterilization cycles.

## Material and Methods

**Figure 1:**
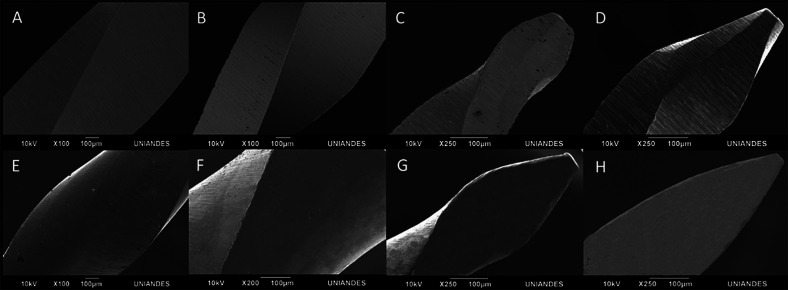
Scanning electron microscope evaluation at 100x and 250x, to verify any signs of visible deformations. (A–D) WOG and (D–F) RB.

G*Power 3.1.9.2 software (Heinrich-Heine-Universität Düsseldorf in Düsseldorf, Germany) was used to estimate the sample size in advance. A total of 210 files, 105 RB “R25” (25/0.08) (VDW, Munich, Germany) and 105 WOG “Primary” (25/0.07) (Dentsply Maillefer, Ballaigues, Switzerland) were evaluated in this in Vitro study. All the instruments were previously examined at different magnifications (each instrument’s active part was captured via multiple images that covered its whole surface), under a scanning electron microscope (JEOL JSM 6490) to detect structural deformations. None of the files had visible defects. Consequently, a dynamic cyclic fatigue test was performed on all of the examined files (Fig. 1).

-Study design

The 210 specimens were randomly assigned into 7 groups as previously described by Pedulla *et al*. (12). Each group was composed of 30 files (WOG (n = 15) and RB (n= 15)) (Supplement 1) 

(http://www.medicinaoral.com/medoralfree01/aop/jced_60870_s01.pdf).

Group G (control group): New files that were not exposed to autoclave sterilization and/or NaOCl immersion before the dynamic cyclic fatigue test.

Group A and D: Files were dynamically immersed for 3 minutes in 5% NaOCl (ENZOHIP – 5, Prodont Scientific, Bogota, Colombia) at 37˚C, 1 and 3 times, respectively. Each file was introduced 16mm deep via dynamic immersion (activated in a VDW Silver Reciproc motor (VDW, Munich, Germany) at the RECIPROC ALL setting) in a glass container filled with 5% NaOCl solution. After removal from NaOCl immersion, files were immediately rinsed with bidistilled water to counteract the effects of NaOCl, dried, assigned an identifying number, and kept in a glass vial until dynamic cyclic fatigue test. Note: In group D, there was a 30 minutes lapse between each dynamic immersion, and files were not rinsed with bidistilled water between immersions, but were promptly rinsed after the last immersion.

Group B and E: Files were autoclaved 1 and 3 times, respectively. Each file was packed individually in sterilization pouches. Every autoclave sterilisation cycle (Classic autoclave; Star clave, Bogota, Colombia) lasted 17 minutes at 134˚C. Files exposed to multiple sterilizations (Group D) were allowed to cool to room temperature and repackaged between each cycle (8,12).

Group C and F: In group C, files were both, dynamically immersed in 5% NaOCl, 1 time and sterilised 1 time (as previously described). In Group F, both protocols (dynamic immersion in NaOCl and autoclaving) were performed 3 times. Autoclaving was performed before NaOCl immersion for both groups.

-Dynamic cyclic fatigue test

**Figure 2:**
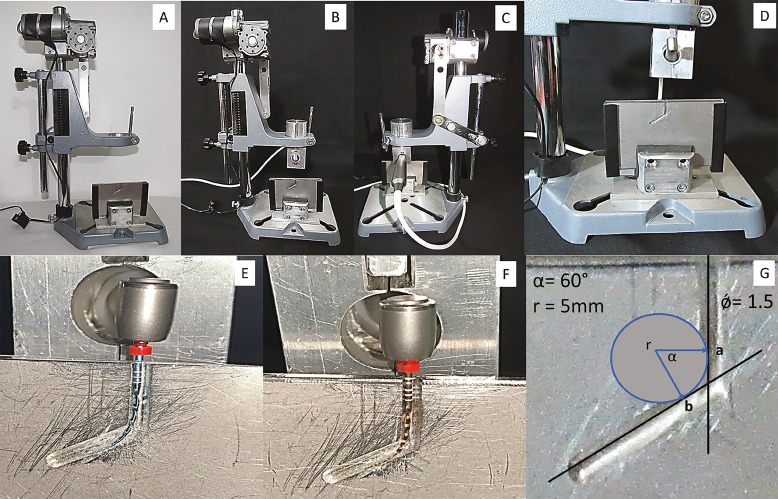
A-C. Dynamic cyclic fatigue testing device, D. 300 series stainless-steel artificial canal, E. RB file during the dynamic cyclic fatigue test, F. WOG file during the dynamic cyclic fatigue test, G. Geometric characteristics of the artificial canal: angle of curvature (α), radius of curvature (r) and internal diameter (ǿ).

A dynamic cyclic fatigue testing device was developed by the endodontics research department of the University Colegio odontologico colombiano UNICOC, according to the criteria previously established by Pruett *et al*. (13). Briefly, the test was conducted in a 300 series stainless-steel artificial canal, with a 5 mm radius (r) of curvature, a 60 ° angle (α) of curvature, and a 1.5 mm continues internal diameter (ǿ) (13). The Radius of curvature and angle of curvature were assessed as previously described by Pruett *et al*. (13) (Fig. 2).

Files from all groups were activated in a VDW Silver Reciproc motor (VDW, Munich, Germany) according to manufacturer’s recommendation, connected to the testing device, which allowed the instruments to freely rotate within the artificial canal under continuous pressure (12). The steel canal was lubricated with synthetic oil to lessen the instruments’ friction. The files were operated with an axial movement of 3 mm/sec until fractured occurred. Files reached a maximum length of 22 mm inside the artificial canal during the cyclic fatigue test (Fig. 2).

**Figure 3:**
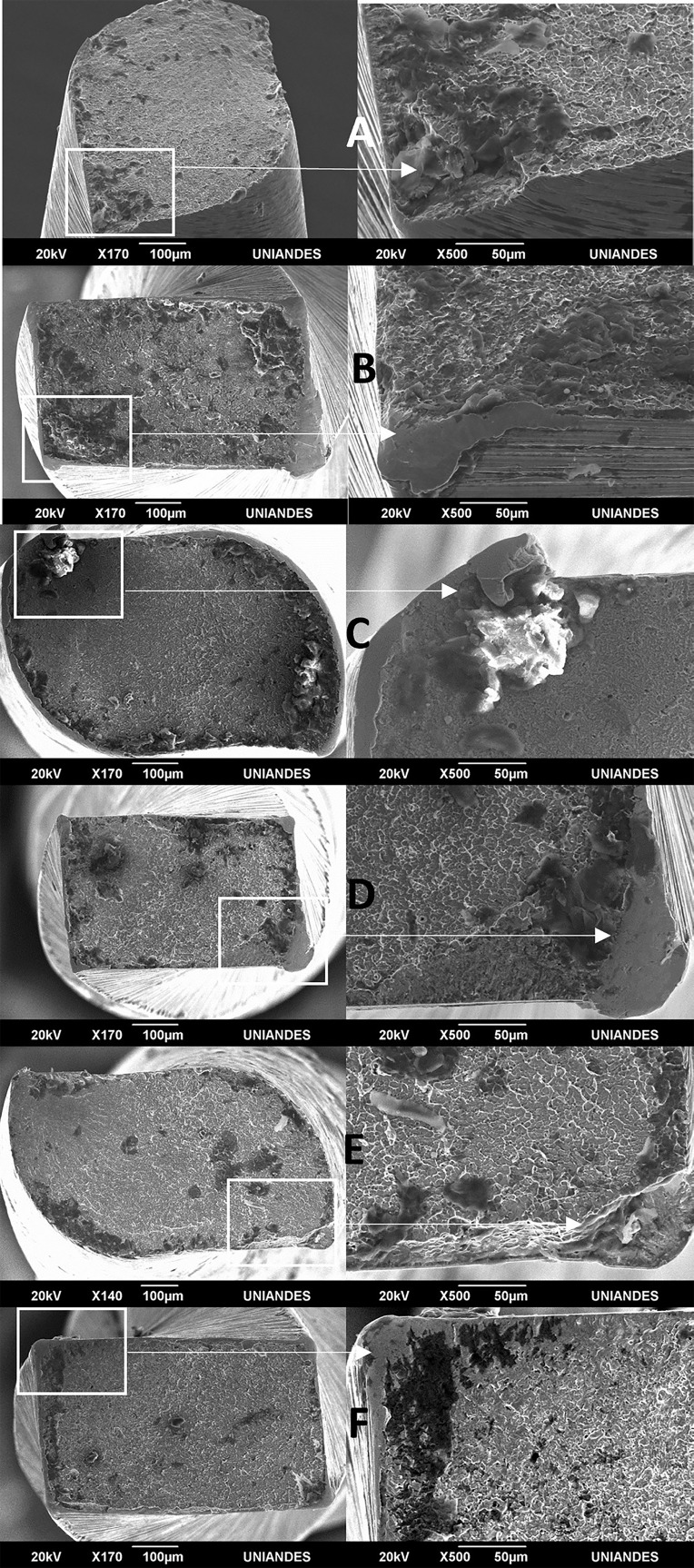
Representative scanning electron microscopic images of fractured specimens (axial views). A-C-E correspond to RB files from groups G, E and F respectively. B-D-F correspond to WOG files from groups G, E and F respectively. On the same surface plane, similar fracture patterns (composed of identifiable brittle and plastic areas) are observed.

A timer was used to record the fracture time for each tested file. In addition, a video record of each instrument was made to cross-check the fracture time. The fractured instruments were analysed under a scanning electron microscope ((JEOL JSM 6490). The accelerating voltage used was 20kV at a working distance of 9.6mm. Photomicrographs of the fractured surfaces were taken at various magnifications (140x to 500x) (Fig. 3).

Before cyclic fatigue test, the chemical composition of the files’ surface from the control group (Group G) was analysed by scanning electron microscope (TESCAN-LYRA 3), equipped with a microanalysis system for energy-dispersive X-ray spectroscopy (OXFORD-XMAX-80 EDS). Dispersive X-ray spectroscopy (EDS) allows obtaining a spectrum with the different chemical elements present in the analysed area, as well as the relative atomic wt % for each of them (AZTEC.3.3 SP1 software). An observation and analysis area of 100µm2 was selected for each file (from the middle region) on 1.78kx magnified images. The effective EDS analysis time was 72 seconds.

-Statistical analysis

Data were subjected to the D’Agostino-Pearson test to check their normal distribution. CFR time was described using means, standard deviation (SD) and frequency (n), in order to test the hypothesis:

H0: X̄A = X̄B =, … , = X̄G

H1: X̄A ≠ X̄B ≠, … , ≠ X̄G

Data were statistically analysed using 1-way and 2-way analyses of variance (ANOVA) and a T-test. The assumption of homoscedasticity was evaluated using the Levene’s test. In case of rejection of H0 a Tukey HSD/Kramer multiple comparison post hoc test was conducted to assess significant differences among groups. Finally, data obtained from EDX analysis were subjected to Student’s t-test to compare the relative wt % of each element between the two brands of files (RB/WOG). A *p-value* ≤0.05 was set to be statistically significant. The statistical analysis was performed with the software STATA V. 17.

## Results

**Figure 4:**
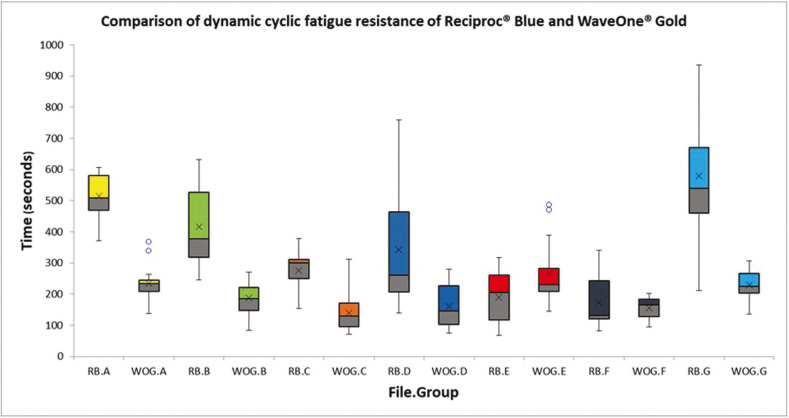
Box Plot analysis of the dynamic cyclic fatigue resistance of RB and WOG after autoclave sterilization and/or immersion in sodium hypochlorite.

Table 1 displays the means and SD of time (expressed in seconds) of the dynamic cycle fatigue test for each group (RB/WOG). Additionally, the graphic comparison of the dynamic CRF of RB and WOG after sterilization and/or immersion in sodium hypochlorite is shown in Figure 4. When the brand of the instruments was considered an independent variable, the inferential analysis showed statistically significant differences between groups (1-way ANOVA, *p*<0.05). When the kind of treatment was considered as the independent variable, there were statistically significant differences between the tested instruments (t-test, *p*<0.05). Moreover, there were significant differences when considering the kind of treatment as a first factor and the brand of the instrument as the second factor (2-way ANOVA, *p*<0.05; interaction <0.05).

**Table 1:**
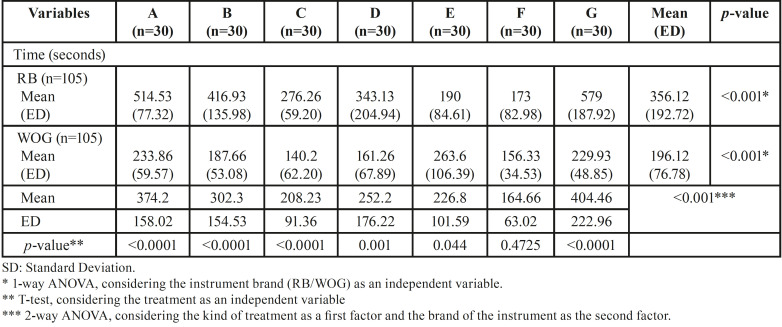
Means and Standard Deviations (SD) of time (expressed in seconds) of the dynamic cycle fatigue test for RB and WOG instruments in all groups.

There was a significantly higher CFR of RB files than WOG in groups A, B, C, D and G (control group) (*p*<0.05). The CFR of WOG files was superior to RB in group E (*p*<0.05). There were no statistically significant differences between files in group F (*p*>0.05) (Table 1).

Post hoc analysis revealed CFR differences between the control and most of the tested groups of RB. Files from groups B, C, D, E and F of RB, had significantly lower resistance than new ones (Group G) (*p*<0.05). Instead, the CRF of RB files was not adversely affected by 3 minutes of immersion in 5% NaOCl for 1 time (Group A) (*p*>0.05) (Table 2).

**Table 2:**
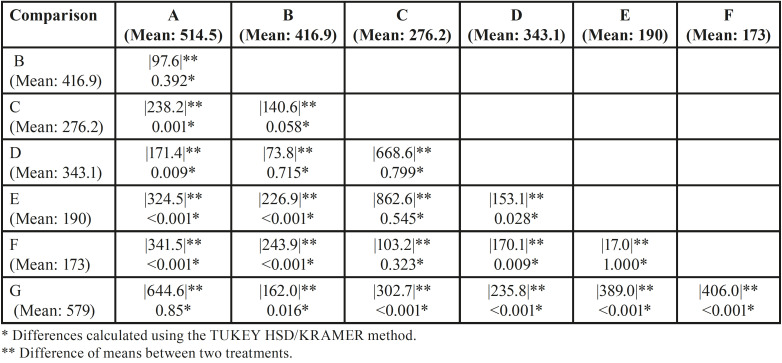
Multiple treatment comparisons for RB instruments.

On the other hand, WOG files exposed to 5% NaOCl in combination with sterilization cycles (Groups C and F) had significantly lower CFR than new ones (*p*<0.05), independent of the number of times (1 or 3 times). No CRF difference was found between groups C and F (*p*>0.05). There were no statistical differences between groups A, B, D and E of WOG and the new ones (Group G) (*p*>0.05) (Table 3).

**Table 3:**
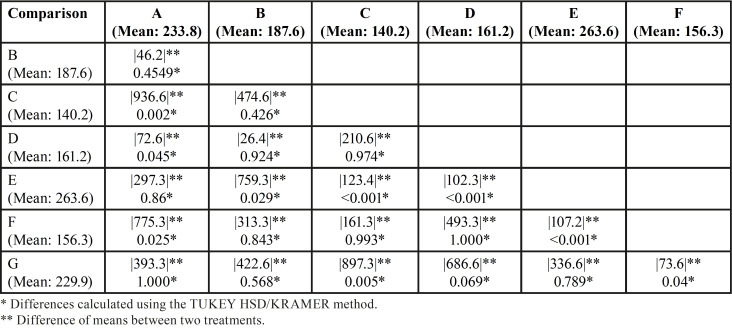
Multiple treatment comparisons for WOG instruments.

The EDS analysis indicated that the chemical elements present in the evaluated areas of both tested instruments (RB/WOG) were similar. However, RB and WOG NiTi alloys differed in atomic wt %. Higher wt % of carbon (C) and oxygen (O) was found in RB alloy compared to WOG alloy (*p*<0.05). Instead, higher wt % of Ni and titanium (Ti) were found in WOG compared to RB (*p*<0.05) (Table 4).

**Table 4:**
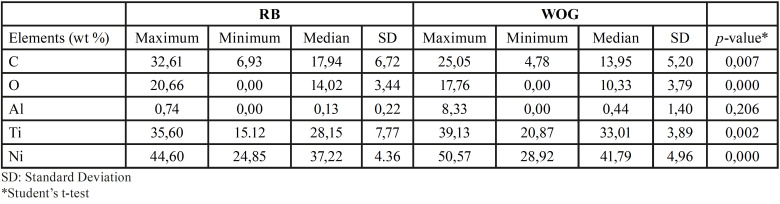
Maximum and minimum values, media and standard deviation (SD) of elemental analysis (EDX) of the external surface of RB and WOG.

## Discussion

Material fatigue appears to be a major cause of NiTi instrument fracture during clinical use (12). Reciprocating instruments have grown in popularity due to their ability to reduce instrument fracture incidence when compared to continuous rotation systems (4). Similarly, manufacturing methods, particularly heat treatments, are crucial for increasing the mechanical properties of endodontic instruments (14). However, there are conflicting results in the literature when comparing cycle fatigue resistance among heat-treated instruments (14). Likewise, though multiple studies have been conducted to evaluate the effects of NaOCl and sterilisation methods on the impact of mechanical properties of classic NiTi rotary instruments, the available evidence on heat-treated NiTi files is minimal, and the results are conflicting (12,14). Therefore, this study analysed the effect of NaOCl and autoclave sterilisation on the CFR of RB and WOG instruments.

A total of 210 new reciprocating files (RB “R25” (n=105) and WOG “Primary” (n=105)) were evaluated in this *in vitro* study. The RB “R25” (25/0.08) instrument attempted to be used in most of the average root canals, displays an S-shaped cross-sectional shape and a non-cutting tip, and features a taper of 0.08% over the first 3 mm that reduces to 0.04% towards the 4th mm, and from the 5th mm until the end of the working part, it keeps a progressive taper of 0.05% (6,7). The WOG Primary file (25/0.07) exhibits a parallelogram-shaped cross-section with alternate one- and two-point contact and a semi-active tip. The primary size, which is thought for preparing most of the canals, features a taper of 0.07% over the first 3mm, which reduces to 0.05% (6,7).

As surface imperfections such as grooves and cracks have been shown to reduce file lifespan in terms of fracture resistance, all instruments were previously examined under scanning electron microscope to rule out structural deformations. To closely mimic clinical settings, the instruments were immersed in 5% NaOCl solution for 3 minutes, 1 (Group A) and 3 (Group D) times (12). There was a 30-minute interval between each dynamic immersion in group D to mimic the average time necessary for performing standard endodontic therapy. To avoid galvanic corrosion, just the 16 mm active part of the instruments were immersed in the NaOCl solution (12). Likewise, to represent the approximate number of times files can be reused, 1 or 3 sterilisation cycles were performed at a temperature of 134˚C for 17 minutes as performed in clinical conditions (12,15). To improve the reliability of cycle fatigue testing, we used artificial canals that allow for the perfect fit and trajectory of the instrument, as well as a dynamic model that allow for better simulation of axial movements in a clinical context (14). Files were operated with an axial movement of 3 mm/sec in an artificial canal with 60˚ angle of curvature and a 5 mm radius of curvature, since most similar previous studies on dynamic cyclic fatigue were based on these parameters (12,14).

In the present study H0 was rejected, since significant differences were found among the tested instruments. In general terms, RB showed higher CFR than WOG files (p≤0.05). This result could be attribuTable to a variety of factors relating to instrument design, particularly the cross-section and metal core mass, as well as thermomechanical processes during file production (16,17). WOG instruments have a parallelogram-shaped cross-section, whereas RB files have S-shaped cross-sections. Several studies have suggested that instruments featuring a greater cross-sectional area and a greater metal core such as the WOG instruments had a significantly reduced CFR (16,18). Besides, the gold thermomechanical treatment has been linked to a 2-stage transformation behaviour (austenite - R-phase - martensite), that results in finely dispersed Ti3Ni4 precipitates in the austenitic matrix and an austenite finish temperature (Af), higher than 50°C, indicating that the alloy undergoes reverse transformation via the R phase (17). Reciproc Blue, on the other hand, exhibits a single-stage transformation (austenite-martensite) and a lower Af (38.4 ± 0.6°C), hence its superior CFR could partially be explained by the presence of a stable martensite phase (19). Furthermore, our EDS analysis revealed that Both files’ surfaces (RB/WOG) had a comparable chemical composition. However, there was a higher wt % of C and O and a lesser wt % of Ni and Ti on the RB surface than on the WOG surface (*p*<0.05) (Table 4). Similar results were reported by Hamdy *et al*. (20). These findings may provide another plausible explanation for the superiority of RB instruments in terms of cycle fatigue resistance. Titanium has two allotropes; hexagonal-closed packed crystalline (α or austenite) or body-centred cubic (β or martensite) forms. Titanium alloying elements are classified as neutral or alphagenic depending on the stabilising effects of the α and β phases. The transition temperature β is raised by alphagenic elements. The most common alphagenic elements are Aluminium (Al), C, and O. Therefore, the presence of these elements in high concentrations as in the case of RB instruments results in a more martensitic crystalline structure, making alloys more flexible and fracture-resistant (21). Notably, WOG instruments outperformed RB instruments only after three sterilisation cycles (Group E), owing to a significant decrease in CFR of RB files compared to their control group (p˂0.05).

It has been proposed that the high temperatures and pressure produced during sterilisation cycles may produce a change in the metallurgical characteristics, due to the alloy’s transition from the martensite phase to the stiffer austenite phase impacting the file’s integrity and flexibility (22). Likewise, autoclaving can corrode the surface of the endodontic files, with a cumulative effect on the structure of the rotary NiTi instruments (23). Furthermore, multiple autoclave cycles can increase the depth of NiTi instruments surface defects, increasing fatigue crack propagation (23). The current study found that RB files sterilised 1 and 3 times, showed a significant decrease in CFR (p˂0.05), proportional to the number of sterilisation cycles. However, these findings are debaTable because previous similar studies concluded that sterilisation cycles had no effect on the CFR of RB files (24,25). These different findings could be caused by the different sterilization protocols followed, the nature of the testing (static or dynamic) and the material on which the test was conducted (resin blocks with simulated root canals or stainless-steel artificial canals). Sterilization cycles (1 or 3 times) did not influence the CFR of WOG files (*p*>0.05).

Corrosion due to interaction with NaOCl, is another factor that may compromise the CFR of NiTi instruments. NaOCl can selectively remove/ dissolve Ni from NiTi alloys, affecting the physical and mechanical properties of endodontic files (26). This pitting corrosive phenomenon of NaOCl occurs due to the anodic current density profile up to -h300 mV typical of NiTi alloys, where the current density does not significantly increase with polarization. Furthermore, in the presence of NaOCl, a reduction in anodic current density and polarity inversion (cathodic to anodic) are suggestive of different corrosion potentials responsible for the surface alterations (9). The current study found that RB files exposed 3 times to dynamic immersion in NaOCl, showed a significant decrease in CFR (*p*<0.05). A single immersion, on the other hand, had no effect on the CFR of RB files. The fact that the detrimental effects of NaOCl on heat-treated NiTi alloys are directly proportional to exposure period can explains these findings (26,27). However, dynamic immersion in NaOCl (1 or 3 times) did not influence the CFR of WOG files (*p*>0.05). The higher wt % of Ni and Ti on the WOG surface than on the RB surface (*p*<0.05) seen in our EDX analysis (Table 4), could partially explain this result. A higher Ni wt % could mitigate the pitting corrosive action (selectively remove/ dissolve Ni) of NaOCl on the surface of WOG instruments. However, contradictory findings demonstrating that, like RB files, immersion of WOG instruments in NaOCl solutions dramatically lowered their cycle fatigue resistance have recently been reported (28,29).

Finally, the lowest levels of CFR were reported when the instruments (RB/WOG) were subjected to the combined treatment of autoclaving and immersion in 5% NaOCl (1 and 3 times), indicating a synergistic impact between the two treatments. Only one previous study, by Kermeoglu *et al*., documented the effect of immersing WOG files in NaOCl combined with sterilisation cycles, and their findings were comparable to ours (30). After sterilization, file exposure to NaOCl may increase the depth of surface irregularity, demonstrating the combined effect of cracking generated by autoclaving and NaOCl-induced corrosion (30). To the best of our knowledge, this is the first study that evaluates the combined effect of autoclaving and NaOCl immersion on RB files.

The major limitation of this study is the in-vitro setting, which does not replicate all of the complex aspects associated with clinical scenarios. The discrepancy in the results observed in the literature might be explained by the diverse methodology in terms of sterilization cycles and immersion procedures used, necessitating additional research to standardise the methods and devices used.

## Conclusions

In general, the RB “R25” files outperformed the WOG “Primary” files in terms of CFR. The RB files, on the other hand, were more vulnerable to the different cycles of immersion in NaOCl or autoclave sterilisation. The combined autoclaving and NaOCl cycles had the most significant impact on the mechanical properties of both files. As both treatments may have a negative impact on the clinical performance of both instruments, and to reduce the danger of instrument fracture, a single use of NiTi rotary files may be advised.
